# Physician Gender, Patient Risk, and Web-Based Reviews: Longitudinal Study of the Relationship Between Physicians’ Gender and Their Web-Based Reviews

**DOI:** 10.2196/31659

**Published:** 2022-04-08

**Authors:** Danish Hasnain Saifee, Matthew Hudnall, Uzma Raja

**Affiliations:** 1 Department of Information Systems, Statistics, and Management Science The University of Alabama Tuscaloosa, AL United States; 2 Department of Systems and Technology Auburn University Auburn, AL United States

**Keywords:** web-based physician reviews, gender, gender bias, patient perception, Alabama, patient risk

## Abstract

**Background:**

Web-based reviews of physicians have become exceedingly popular among health care consumers since the early 2010s. A factor that can potentially influence these reviews is the gender of the physician, because the physician’s gender has been found to influence patient-physician communication. Our study is among the first to conduct a rigorous longitudinal analysis to study the effects of the gender of physicians on their reviews, after accounting for several important clinical factors, including patient risk, physician specialty, and temporal factors, using time fixed effects. In addition, this study is among the first to study the possible gender bias in web-based reviews using statewide data from Alabama, a predominantly rural state with high Medicaid and Medicare use.

**Objective:**

This study conducts a longitudinal empirical investigation of the relationship between physician gender and their web-based reviews using data across the state of Alabama, after accounting for patient risk and temporal effects.

**Methods:**

We created a unique data set by combining data from web-based physician reviews from the popular physician review website, RateMDs, and clinical data from the Center for Medicare and Medicaid Services for the state of Alabama. We used longitudinal econometric specifications to conduct an econometric analysis, while controlling for several important clinical and review characteristics across four rating dimensions (helpfulness, knowledge, staff, and punctuality). The overall rating and these four rating dimensions from RateMDs were used as the dependent variables, and physician gender was the key explanatory variable in our panel regression models.

**Results:**

The panel used to conduct the main econometric analysis included 1093 physicians. After controlling for several clinical and review factors, the physician random effects specifications showed that male physicians receive better web-based ratings than female physicians. Coefficients and corresponding SEs and *P* values of the binary variable *GenderFemale* (1 for female physicians and 0 otherwise) with different rating variables as outcomes were as follows: *OverallRating* (coefficient –0.194, SE 0.060; *P*=.001), *HelpfulnessRating* (coefficient –0.221, SE 0.069; *P*=.001), *KnowledgeRating* (coefficient –0.230, SE 0.065; *P*<.001), *StaffRating* (coefficient –0.123, SE 0.062; *P*=.049), and *PunctualityRating* (coefficient –0.200, SE 0.067; *P*=.003). The negative coefficients indicate a bias toward male physicians versus female physicians for aforementioned rating variables.

**Conclusions:**

This study found that female physicians receive lower web-based ratings than male physicians even after accounting for several clinical characteristics associated with the physicians and temporal effects. Although the magnitude of the coefficients of *GenderFemale* was relatively small, they were statistically significant. This study provides support to the findings on gender bias in the existing health care literature. We contribute to the existing literature by conducting a study using data across the state of Alabama and using a longitudinal econometric analysis, along with incorporating important clinical and review controls associated with the physicians.

## Introduction

### Background

Web-based reviews of physicians have been gaining significant popularity among health care consumers or patients over the past 2 decades. Some examples of popular websites for web-based physician reviews are RateMDs [1], Vitals [2], and HealthGrades [3]. The prominence of these reviews is enhanced as the health care landscape in the United States becomes more patient-centric. Patients are becoming more involved in the management of their own health care. Although the review websites were initially popular among certain demographics [4], over time, they have gained significant popularity across a substantial portion of patient population. In fact, a recent survey of web-based physician reviews found that approximately 95% of the respondents viewed web-based reviews to be *somewhat reliable* or *very reliable*, and approximately 70% of respondents said that their choice of a physician was affected by the ratings or reviews on web-based physician review websites [5].

The literature on web-based reviews of physicians has been growing in the past 10 years. Using data from the United States and other countries, numerous studies have examined the content and valence of web-based physician or hospital reviews and the factors that could explain their variance [6-15]. A substream of this literature examined the relationship between the clinical outcomes or performance of physicians and their web-based reviews. The results were quite mixed [13]. Some studies have found a statistically significant association between physicians’ clinical performance and their web-based reviews [16-18]. On the other hand, some studies have found that physicians with better clinical practices or outcomes do not receive better web-based reviews [19-21].

Another substream has investigated the influence of web-based physician reviews on patients’ choices. There has been a significant interest among health care researchers and practitioners in the health care consumers’ awareness of web-based physician reviews [22]. Several studies have investigated whether web-based physician reviews impact patients’ choices and whether there are certain characteristics of these reviews that impact the choice. These studies found that high number of reviews and high valence of reviews were associated with a more positive attitude toward the rated physicians and their selection by patients [23-25].

The increasing reliance on web-based physician reviews is indicated by other surveys also [26]. These surveys of web-based reviews also reveal that a significant portion of patients checks the web-based reviews of physicians, even if they were referred to these physicians by their health care providers. Collectively, these findings reveal the extent to which web-based reviews of physicians have become prominent among patients or health care consumers.

As web-based health care information, including physician reviews, is publicly available and easily accessible, there has been a long-standing concern among the health care providers and research communities about the quality and clinical relevance of web-based health care information [27]. The interaction between health care providers and their patients can affect the patients’ opinions of them. In turn, these opinions can become web-based reviews that are accessible to anyone searching for their physicians’ information on the web.

There has been a long-established interest among researchers in the impact of physician gender on patient communication and patients’ choice of physicians. Extant literature has found that female physicians tend to engage in patient-centered communication [28-30] and do not receive ratings as high as their male counterparts [31,32]. It has also been proposed that the relationship between physicians and their patients might be affected by the physician’s gender and different expectations of patients from male and female physicians [33-35]. The dynamics of patients’ communication and relationship with physicians of different genders have received significant attention in the extant literature [36,37].

Questions about whether patients have a preference for male physicians over female physicians, and vice versa, and whether their opinions of physicians are affected by the physicians’ gender have also received substantial attention from health care professionals and researchers. For instance, in a survey of 185 patients, Fennema et al [38] found that 43% of women and 12% of men preferred a female physician, whereas 31% of men and 9% of women preferred a male physician and that patients who preferred male physicians reported technical competence to be a more prominent characteristic of male physicians. In a different survey, Kerssens et al [39] did not find a preference for surgeons or anesthesiologists of a particular gender, but found preferences for female physicians as gynecologists in 8.5:1 ratio and general practitioners in 2.32:1 ratio among female respondents. In another survey of 125 women, Plunkett et al [40] found that the gender of a physician was not of primary importance when selecting an obstetrician or gynecologist. Some of these studies have also attempted to identify the mechanisms that may have led to their findings. There have also been calls for suggestions on making health care workplaces more equitable for female physicians [41].

With the proliferation of web-based physician reviews among patients or health care consumers, a natural and important question is, “Whether and to what extent is a physician’s gender related to their online reviews after accounting for patient risk and time shocks (time fixed effects)?”

After a careful review of the existing literature, we found that the potential effect of physician gender on web-based reviews of physicians has not received sufficient attention. In the few studies that have examined the relationship between physicians’ gender and their web-based reviews, the findings have been mixed. For example, Dunivin et al [32] and Thawani et al [42] found that female physicians receive lower ratings than male physicians. On the other hand, Emmert and Meier [43] found that female physicians receive better ratings than their male counterparts. Marrero et al [44] found that female surgeons receive more positive ratings for social interaction, whereas male surgeons receive better ratings for technical aspects. Clearly, the possible effect of physicians’ gender on their web-based reviews, or lack thereof, requires more thorough examination.

In the examination of the aforementioned relationship, it is important to account for the characteristics of patients, such as patient risk, in some form. It is also important to account for the variation in the reviews over time to determine the direct relationship between physicians’ gender and their web-based reviews. Including patient risk allows us to account for the health characteristics of a significant patient population under the care of physicians. Not controlling for such characteristics can potentially bias the results because a physician’s interaction can be affected by the existing health condition of their patients. Therefore, we examine the effect of physician gender on web-based patient reviews, while controlling for patient health risks over time.

### Objective

To the best of our knowledge, our study is among the first to examine the effect of physicians’ gender on their web-based reviews over time and after accounting for patient risk. Furthermore, our study is the first to conduct such an investigation using physician data across Alabama, a state that has received very little attention in the literature on web-based physician reviews. We accomplish our analysis by using a unique data set that we created by combining data from web-based physician reviews from a popular physician review website, RateMDs, and clinical data from the Center for Medicare and Medicaid Services (CMS) for the state of Alabama.

## Methods

### Ethics Approval

No ethics board review or approval was required for this study. All the raw data that were collected for this study are publicly available on the web.

### Data

To study whether web-based reviews of physicians are more favorable toward male or female physicians, we constructed a panel data set of physicians in Alabama using data from 2 sources. The unit of analysis in our study was a physician, and the time periods in the panel were years. We collected data on web-based reviews and the gender of physicians from RateMDs to construct our web-based review data set spanning from 2012 to 2018. We used Python (Python Software Foundation) to collect data from RateMDs. We also obtained clinical data on physicians from Medicare Provider Utilization and Payment Data: Physician and Other Supplier [45], which traversed the same time frame of 2012 to 2018. We combined the data from these sources using a combination of physicians’ first names, last names, specialty, and years. Our final unbalanced panel data set had 1093 matching physicians over a 7-year time span (2012 to 2018) that matched both data sets. There were 5912 physicians in the RateMDs data set who had at least one review, and there were a total of approximately 26,600 reviews across these physicians. Among these 5912 physicians, 2673 (45.21%) physicians had reviews in at least two years. We were able to match 40.89% (1093/2673) of these physicians with our data from CMS, and this 40.89% (1093/2673) of the physicians constituted the panel used to conduct the panel analysis in this study.

**Figure 1:**
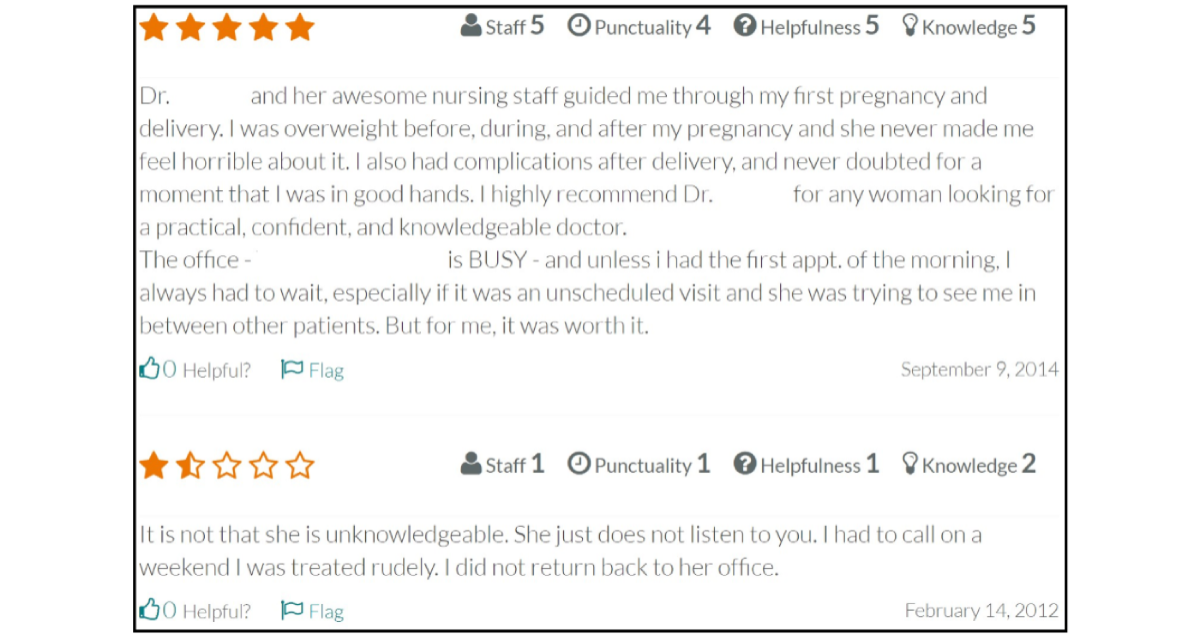
Example screenshot of RateMDs reviews for a physician.

Each physician in our final panel has a unique national provider identification number that was collected from CMS. This ensured that all the physicians in our final panel were unique. Figure 1 shows an anonymized selection of reviews from RateMDs for a physician in our data set. As shown in Figure 1, a physician can receive numeric ratings on four different dimensions (staff, punctuality, helpfulness, and knowledge). Along with these numeric ratings, a physician can also receive textual comments. The dates on which the reviews were provided on RateMDs is also shown in Figure 1. Patient reviews on RateMDs and optional responses by the physicians are free of charge. Paid tiers for physicians exist on RateMDs, but they do not allow for the alteration of reviews. The paid tiers allow for physicians to be notified of new ratings, the ability to feature a rating, appointment requests, photos, and other features, but no paid feature inhibits the ability of a person to post a review on the site.

### Measures

As we were examining whether the web-based reviews of physicians are favorable to male or female physicians, we constructed our dependent variables using the numeric physician ratings from RateMDs. Physicians on RateMDs can be rated on four dimensions: helpfulness, knowledge, staff, and punctuality. The ratings for each of these dimensions are on a scale of 1 to 5, with 5 being the best possible score and 1 being the lowest score. To capture the information in each of these four dimensions, we constructed the following four dependent variables: *HelpfulnessRating*, *KnowledgeRating*, *StaffRating,* and *PunctualityRating*. *HelpfulnessRating* was the average of the ratings received by a physician on the helpfulness dimension in a year. Similarly, *KnowledgeRating*, *StaffRating,* and *PunctualityRating* were the averages of the ratings received by a physician on the knowledge, staff, and punctuality dimensions, respectively. To capture the combined information across these dimensions, we constructed a panel variable, *OverallRating*. For this purpose, initially, we constructed a variable *NetRating* using the average of the ratings received on the four aforementioned dimensions. Then, we constructed *OverallRating* by calculating the average of *NetRating* in each year, similar to how we constructed *HelpfulnessRating*, *KnowledgeRating*, *StaffRating,* and *PunctualityRating*.

Our key explanatory variable was a time-invariant variable, *GenderFemale,* which equals 1 for female physicians and 0 for male physicians. We obtained data on the gender of the physicians from RateMDs. We also used several control variables to account for the clinical aspects associated with the physician and with the textual comments that go alongside numeric *RateMDs* ratings. Our control variables included *RiskScore*, *TopicCare*, *TopicSurgery*, *TopicStaff,* and *Specialty*.

*RiskScore* was the average yearly hierarchical condition category (HCC) risk score calculated by CMS using data on Medicare beneficiaries [45]. HCC coding can provide information about patient complexity and a description of the medical complications a patient is experiencing. HCC relies on the International Classification of Diseases–10th Edition coding to assign risk scores to patients [46]. A physician with high *RiskScore* would have Medicare beneficiaries with high risk scores (above-average spending). This variable allowed us to control for the patient risk score of the Medicare patients under the care of a physician. As Medicare is one of the largest health care insurers or payers in the United States, *RiskScore* helped us to account for the patient risk of a significant proportion of the patient population under the care of physicians.

*TopicCare* was the proportion of textual reviews received by a physician each year, in which the dominant underlying theme was care provided by the physician. *TopicStaff* was the proportion of textual reviews in which the dominant underlying theme was the office or staff of the physician. *TopicSurgery* was the proportion of textual reviews in which the dominant underlying theme was the surgical proficiency of the physician.

To construct these topics (latent topics), we used topic modeling techniques based on Latent Dirichlet Allocation (LDA) [47,48]. LDA has been used extensively for topic modeling in the extant literature on web-based reviews of products and services, including several studies involving web-based physician reviews [9,49-53]. The following sections provide a brief description of the main steps through which we used topic modeling to construct the aforementioned topic variables. We used R (R Foundation for Statistical Computing) for topic modeling.

We created a corpus of all the reviews using an R text-mining package(TM) within RStudio, after which we converted the corpus to lower case [54-56]. We also replaced punctuation, numbers, and stop words. We *stemmed* the corpus to allow us to reduce words with a common root to the root word, such as *nurse* and *nursing* to the word fragment *nurs*. Next, we created the *document-term matrix,* which stored the frequencies of stemmed words in our textual comment corpus by each textual comment. Then, we leveraged the LDA algorithm and used an R package (topicmodels) to extract topics from our textual comments [57-59]. These R packages have been widely used in the literature mentioned previously to construct latent topics or themes from textual data. For each comment, a probability was assigned to each of the identified latent themes or topics, and the probabilities summed up to 1 for each comment. We classified each comment based on the topic that had the highest probability. We identified the most common words within each of the 3 target latent topics, as shown in Textbox 1. We chose these 3 topics because it was the minimum number of topics that we could use to clearly categorize the experiences with the physicians and their staff reported in textual reviews [20,21]. Textbox 1 shows the stemmed words most closely (probabilistically) associated with each of the 3 review comment topics.

**Most prominent words (after stemming) by topic.**

**TopicCare**

- care, doctor, staff, recommend, patient, time, knowledg, help, friend, love, wonder, high, listen, excel, and feel

**TopicStaff**

- time, office, doctor, wait, staff, patient, appoint, call, nurs, rude, visit, day, question, hour, and talk

**TopicSurgery**

- doctor, surgeri, pain, care, medic, life, patient, treat, recommend, time, day, surgeon, procedur, treatment, and feel

We had physicians from across 34 specialties in our final panel data set. The 15 specialties with most physicians (in descending order of the number of physicians) were as follows: general (family) practice, obstetrics and gynecology, internal medicine, orthopedic surgery, neurology, otolaryngology, cardiology, ophthalmology and optometry, psychiatry, dermatology, general surgery, podiatry, urology, endocrinology, and rheumatology. Physicians in these 15 specialties accounted for approximately 85.73% (937/1093) of all the physicians in our panel data set. Table S1 in Multimedia Appendix 1 lists the number of male and female physicians across specialties in the panel data set. The physician specialties were time-invariant binary variables. Controlling for the specialties allowed us to compare the effect of the physicians’ gender on their reviews after accounting for the numerous unobservable time-invariant clinical aspects that could influence physicians of both genders within each specialty. We also conducted further robustness checks by including additional clinical review control variables. These control measures helped us significantly distinguish our research from previous studies.

### Analysis

We used physician random effects panel regression, along with year fixed effects to account for time shocks. A time shock in the context of this paper can be considered as an event or collection of events that can impact physicians across the board in the duration of a year. For example, a statewide or nationwide health care policy change would likely have an impact on physicians across different specialties. As the analysis used panel data, it was important to account for such time shocks. We did so by including year fixed effects in our regression specifications. We used Stata (StataCorp) for conducting our econometric analysis.

We leveraged the physician random effects model instead of the physician fixed effects model to estimate the effect of physician gender because of the following reasons: (1) our main explanatory variable, *PhysicianGender,* was time-invariant, and physician fixed effects would have subsumed the *PhysicianGender* variable and (2) a physician’s gender can be safely assumed to be randomly assigned in the context of our study, and thus, it was very unlikely that there were unobserved variables that could simultaneously drive or influence both the physician gender and their web-based reviews. The year fixed effects allowed us to account for the time shocks in the health care industry or web-based physician review websites that can influence physicians across the state of Alabama. The SEs shown in all the panel regression specifications were robust. For brevity, we do not report the coefficients, SEs, and *P* values of the different specialties and year fixed effects. The sum of *TopicCare*, *TopicSurgery,* and *TopicStaff* was equal to 1. In our specifications, *TopicStaff* was the base topic variable, and thus, not included in the regressions. One of the specialties and one of the years acted as the base specialty and base year, respectively, and thus, were not included in the regression specifications.

## Results

### Descriptive Statistics

**Figure 2:**
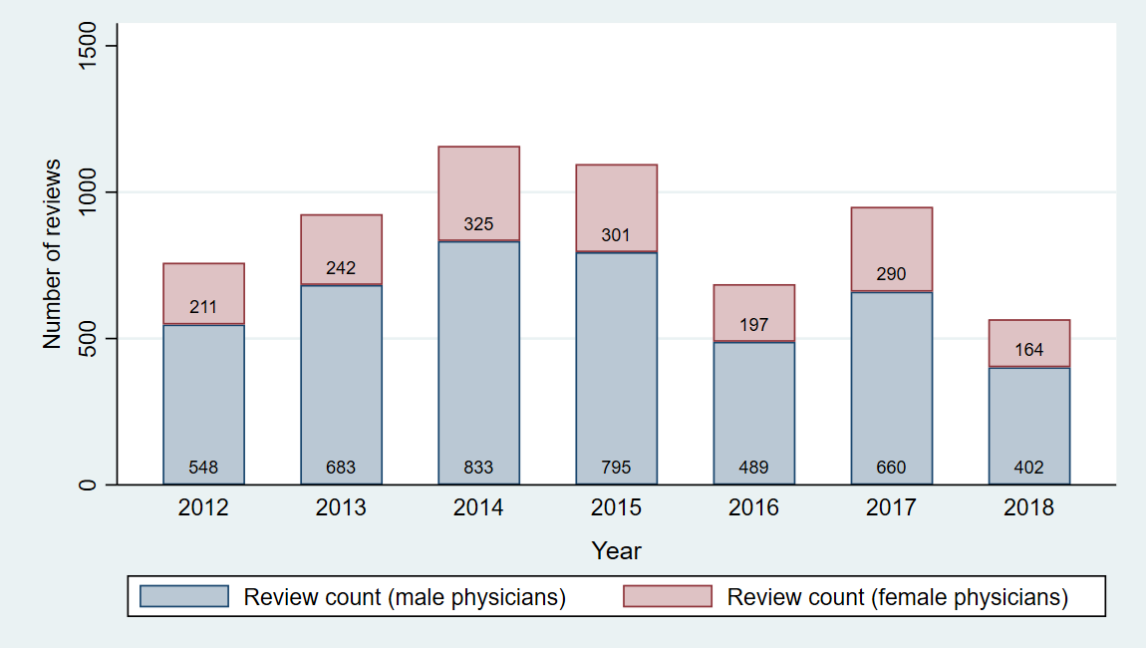
Distribution of total number of physician reviews across years.

Figure 2 shows the distribution of the number of reviews for male and female physicians across the years from the original RateMDs data set. This chart and the subsequent figures were created using the 1093 physicians who were present in our panels across CMS and RateMDs data used for panel regressions. Our panel consisted of a 7-year period spanning from 2012 to 2018 to include a broad set of historical data that were also relatively current. As shown in Figure 2, the year 2014 had the highest number of reviews, whereas 2018 had the lowest number of reviews across the physicians in our panel, and there were ample number of physician reviews across all years in our panel.

**Figure 3:**
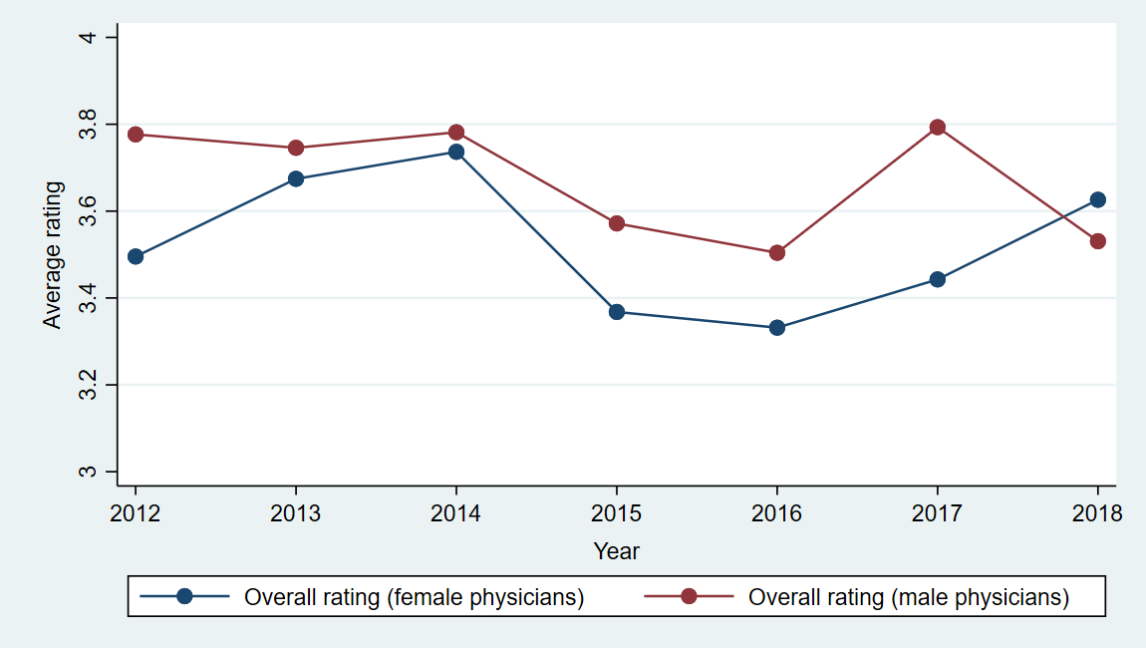
Comparison of average overall ratings for female and male physicians across years.

**Figure 4:**
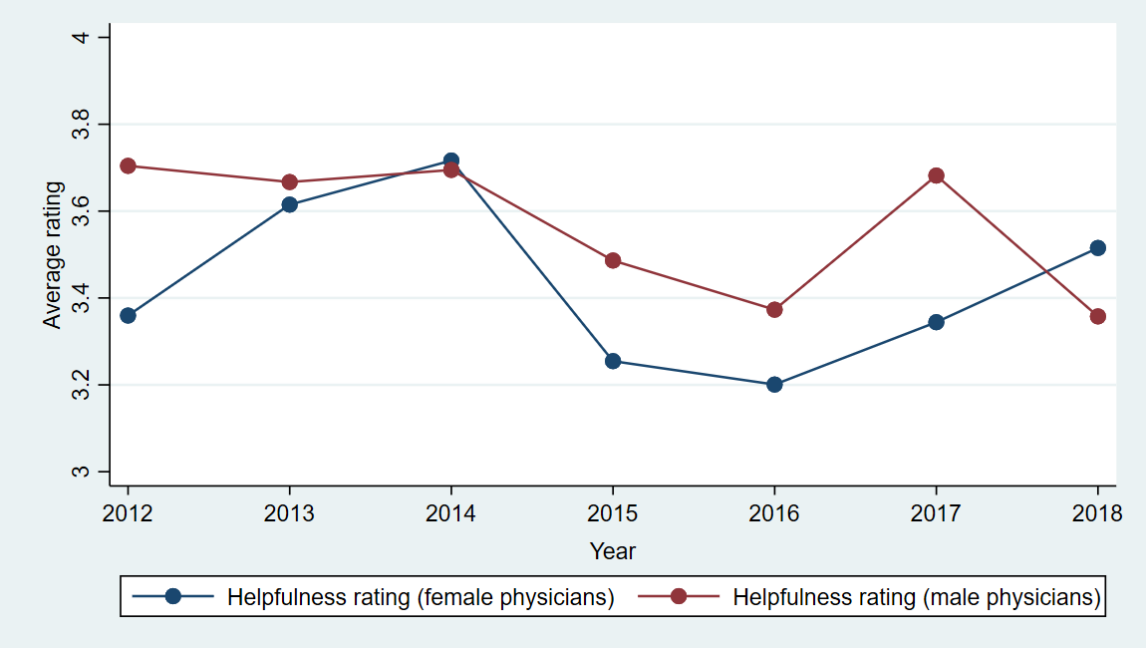
Comparison of average helpfulness ratings for female and male physicians across years.

**Figure 5:**
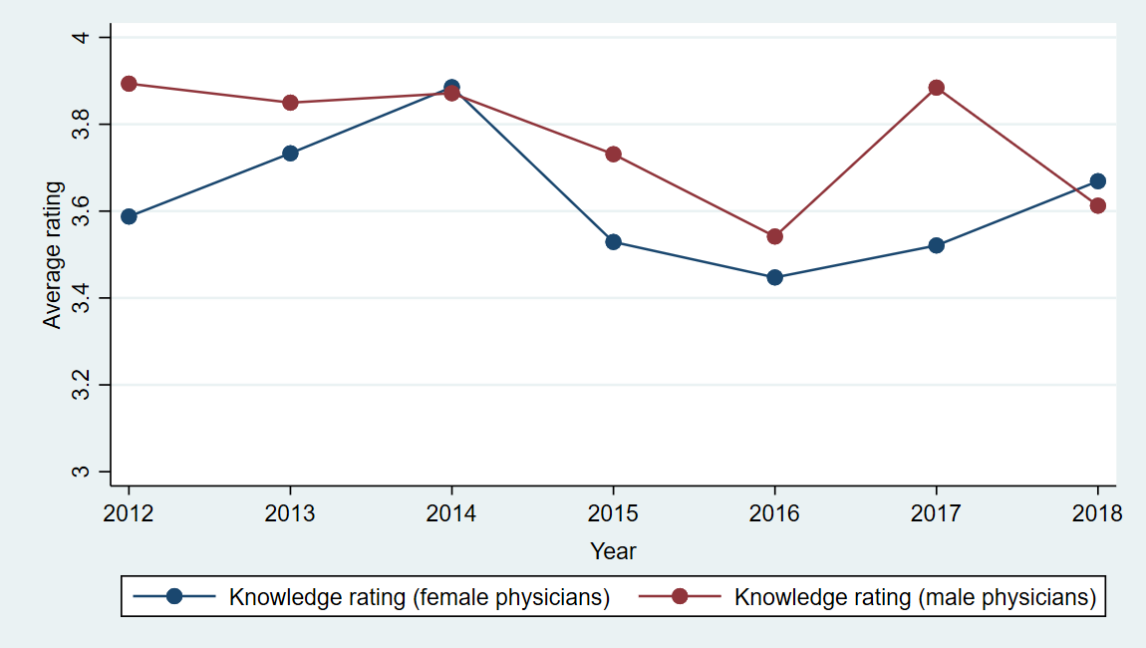
Comparison of average knowledge ratings for female and male physicians across years.

**Figure 6:**
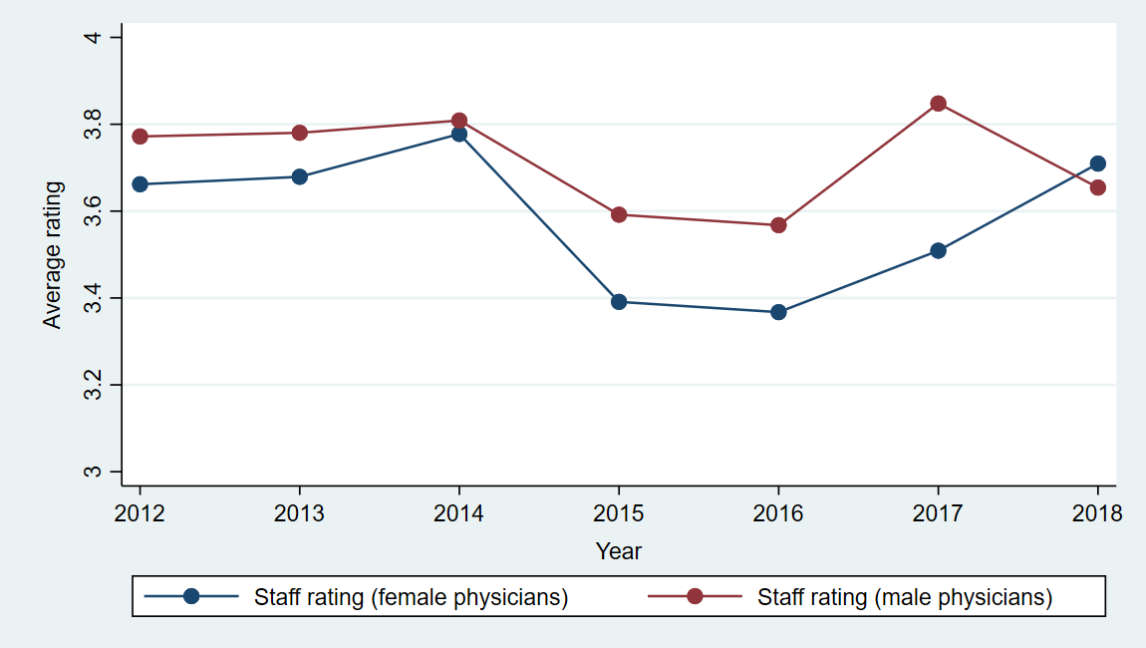
Comparison of average staff ratings for female and male physicians across years.

**Figure 7:**
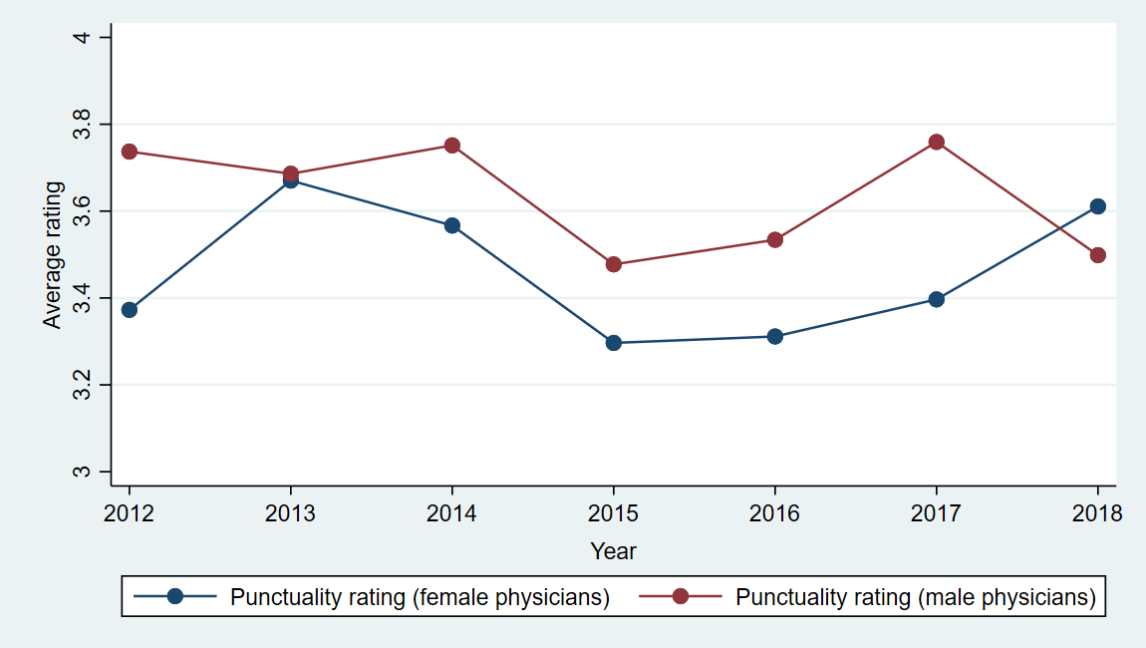
Comparison of average punctuality ratings for female and male physicians across years.

Figures 3-7 show plots of the average annual values of *OverallRating*, *HelpfulnessRating*, *KnowledgeRating*, *StaffRating,* and *PunctualityRating* for male and female physicians for the 1093 physicians from RateMDs who were in the panel. As shown in Figure 3, the average *OverallRating* of male physicians was consistently higher than that of female physicians. The average annual ratings on all 4 dimensions were more favorable for male physicians across most years. The time trends depicted in these figures revealed reviews more favorable toward male physicians than female physicians. The variation in the difference in the average values is visible in these figures and warrants a thorough longitudinal investigation of the effect of physician gender on the web-based ratings. Accordingly, we conducted a longitudinal or panel empirical investigation of the effect of a physician’s gender on their web-based ratings. As stated previously, we controlled for several clinical and review characteristics associated with physicians, and by doing so, we isolated the direct effect of a physician’s gender on their web-based ratings.

Table 1 shows the descriptive statistics of the various dependent variables, topic controls, and *RiskScore* control. The average values of the rating variables were between 3.5 and 3.6. In our panel, approximately 25.34% (277/1093) of the physicians were women. In Alabama, female physicians account for approximately 28.5% (3025/10,614) of the overall physician population [60]. This suggests that the overall distribution of physician gender in our panel was fairly representative of that in Alabama.

**Table 1:** Descriptive statistics (number of observations=3446).

Variable	Values, mean (SD)	Values, median	Values, minimum	Values, maximum
*OverallRating*	3.64 (1.43)	4.25	1	5
*HelpfulnessRating*	3.54 (1.65)	4.37	1	5
*KnowledgeRating*	3.74 (1.54)	5	1	5
*StaffRating*	3.69 (1.48)	4	1	5
*PunctualityRating*	3.60 (1.49)	4	1	5
*TopicCare*	0.41 (0.45)	0	0	1
*TopicSurgery*	0.27 (0.40)	0	0	1
*TopicStaff*	0.32 (0.42)	0	0	1
*RiskScore*	1.23 (0.41)	1.14	0.53	5.62

### Effect of Gender

Table 2 provides the results of random effects panel regression, with *OverallRating* as the dependent variable. We included physician specialties as controls and year fixed effects in each of the regression specifications. The SEs of each specification were robust. As shown in Table 2, the coefficient of *GenderFemale* was negative and statistically significant, implying that female physicians tend to receive worse overall web-based ratings than their male counterparts. The coefficient of *RiskScore* was statistically insignificant in all the specifications, implying that physicians who treat Medicare patients of high risk tend to not receive better or worse overall ratings than their counterparts who treat Medicare patients of low risk. The coefficients of *TopicCare* and *TopicSurgery* were positive and statistically significant, implying that the physicians who receive a high proportion of review comments with an underlying theme of physician care and surgical aspects tend to have better overall ratings than those who receive a high proportion of review comments with an underlying theme of their office and staff. In Tables 3 and 4, the coefficient of *GenderFemale* was negative and statistically significant for *HelpfulnessRating*, *KnowledgeRating,* and *PunctualityRating,* but not for *StaffRating*.

The coefficient of *RiskScore* was statistically insignificant for each of the four rating dimensions, whereas that of *TopicCare* and *TopicSurgery* were positive and statistically significant. The magnitude of the coefficient of *GenderFemale* was close to 0.2. This means that, on average, female physicians receive ratings lower by 0.2 points than their male counterparts. For example, on average, if male physicians receive a rating of 4 out of 5, their female counterparts would receive a rating of 3.8 out of 5.

**Table 2:** Estimation for OverallRating (N=1093)^a^.

Variable	Coefficient (SE)	*P* value
*GenderFemale*	−0.162 (0.060)	.007
*RiskScore*	−0.056 (0.086)	.52
*TopicCare*	1.557 (0.058)	<.001
*TopicSurgery*	0.739 (0.071)	<.001

^a^Specialty controls=yes; year fixed effects=yes; robust SE=yes; overall R-squared=0.267; within R-squared=0.168; between R-squared =0.339.

**Table 3:** Estimation for HelpfulnessRating and KnowledgeRating (N=1093).

Variable	*HelpfulnessRating* ^a^			*KnowledgeRating* ^b^	
	Coefficient (SE)	*P* value	Coefficient (SE)		*P* value
*GenderFemale*	−0.185 (0.069)	.008	−0.198 (0.065)		.002
*RiskScore*	0.003 (0.098)	.97	−0.057 (0.094)		.54
*TopicCare*	1.702 (0.069)	<.001	1.492 (0.064)		<.001
*TopicSurgery*	0.688 (0.084)	<.001	0.513 (0.080)		<.001

^a^Specialty controls=yes; year fixed effects=yes; robust SE=yes; overall R-squared=0.239; within R-squared=0.153; between R-squared=0.310.

^b^Specialty controls=yes; year fixed effects=yes; robust SE=yes; overall R-squared=0.220; within R-squared=0.137; between R-squared=0.282.

**Table 4:** Random effects panel regression (StaffRating and PunctualityRating; N=1093).

Variable	*StaffRating* ^a^			*PunctualityRating* ^b^	
	Coefficient (SE)	*P* value	Coefficient (SE)		*P* value
*GenderFemale*	−0.095 (0.062)	.13	−0.172 (0.067)		.01
*RiskScore*	−0.045 (0.087)	.61	−0.127 (0.105)		.23
*TopicCare*	1.547 (0.063)	<.001	1.488 (0.063)		<.001
*TopicSurgery*	0.923 (0.076)	<.001	0.832 (0.074)		<.001

^a^Specialty controls=yes; year fixed effects=yes; robust SE=yes; overall R-squared=0.247; within R-squared=0.155; between R-squared=0.315.

^b^Specialty controls=yes; year fixed effects=yes; robust SE=yes; overall R-squared=0.234; within R-squared=0.130; between R-squared=0.318.

### Robustness Checks

We added additional control variables to check whether our findings would change. The three additional variables were *BeneficiaryCount*, *ServicesCount,* and *WordCount*. *BeneficiaryCount* was the number of Medicare beneficiaries under the care of a physician in a year. *ServicesCount* was the number of services provided by a physician in a year. *WordCoun*t was the average number of words in the review comments received by a physician in a year. Tables S2-S4 in Multimedia Appendix 1 provide the results of panel specifications with additional control variables. Table S2 in Multimedia Appendix 1 provides the results with *OverallRating* as the dependent variable. Table S3 in Multimedia Appendix 1 provides the results with *Helpfulness* and *KnowledgeRating* as the dependent variables, and Table S4 in Multimedia Appendix 1 provides the results with *StaffRating* and *PunctualityRating* as the dependent variables. As can be observed in Tables S2-S4 in Multimedia Appendix 1, the coefficients *of GenderFemale* were negative and statistically significant for *OverallRating* and each of the four rating dimensions, including *StaffRating*. The magnitude of coefficient of *GenderFemale* was close but slightly higher than those in Tables 2-4.

We conducted further robustness checks by removing the specialties in our panel in which both genders were not represented. This helped us mitigate the concern that a possible bias may arise owing to the absence of physicians of one of the genders in any of the specialties in our panel. The results displayed in Tables S5-S7 in Multimedia Appendix 1 are consistent with our original findings that female physicians receive lower ratings than their male counterparts.

In our next robustness check, we conducted our main regression analysis without topic controls. This test was conducted to examine whether the topic variables may have introduced a systemic bias in the specifications owing to the manner in which they were constructed and whether the negative coefficient of *GenderFemale* variable may have been an artifact. As can be observed from the results in Tables S8-S10 in Multimedia Appendix 1, the coefficient of *GenderFemale* was negative and statistically significant across the specifications, even after topic controls were excluded. This further supports our main finding that female physicians tend to receive worse web-based reviews than their male counterparts. The topic controls play an important role in our specifications because they help to explain part of the variance in the web-based ratings. This can be further understood by comparing the overall R-squared, within R-squared, and between R-squared values in Tables 2, 3, and 4 with those in Tables S8, S9, and S10 in Multimedia Appendix 1, respectively. The 3 R-squared values were substantially higher in Tables 2-4, which means that the topic controls explained a considerable part of the variance in the web-based rating variables.

In summary, we conducted three additional robustness checks as explained above: (1) included additional control variables, (2) removed the specialties that did not include physicians of both genders, and (3) removed the topic controls. After conducting these robustness checks, we can conclude that female physicians tend to receive worse web-based reviews than their male counterparts. This finding is consistent across the regression specifications used in this study.

A concern could be about how representative the data in our panel are of the original data collected from RateMDs and Medicare (CMS). To address this concern, we calculated the descriptive statistics of the variables shown in Table 1 using the original longitudinal data collected from RateMDs and Medicare. The descriptive statistics are shown in Table S11 in Multimedia Appendix 1. A comparison of the statistical values in Table S11 in Multimedia Appendix 1 shows that the panel data used for the econometric analysis in our study are fairly representative of the original data collected from the 2 aforementioned sources.

## Discussion

### Overview

Our study provides an important contribution to the growing literature on web-based physician reviews and physician gender. A possible concern could be that the differences observed in the reviews between physicians of different genders could be driven by the differences in the quality of care or outcomes delivered by physicians of different genders. To address this concern, we performed a substantial search of the existing literature examining the differences between the quality of clinical care or outcomes delivered by male and female physicians. We found several research papers in this context [61-65], but we could not find significant evidence from extant research that male physicians deliver better care than female physicians.

### Principal Findings

We found that male physicians receive better web-based reviews than female physicians after controlling for their clinical characteristics such as specialty and patient risk. Although the difference between the web-based ratings for male and female physicians was statistically significant, the average magnitude of the difference was not substantial. Our findings support that of Dunivin et al [32] and Thawani et al [42], but do not support the findings of Emmert and Meier [43], who found that during the examined time frame, female physicians had better reviews than male physicians. Their results indicated a slight but statistically significant preference for female physicians (2% differential in the percentage of reviews below the mean for each gender) compared with our results that found a 0.2 differential on a 5-point scale in favor of male physicians (4% difference). Possible reasons for these differences could be attributed to cultural variations between the patient populations in Alabama and Germany and that the reviews collected by Emmert and Meier [43] included more female respondents than male respondents. It is also possible that the relationship between patients and their physicians were not in favor of male physicians in Germany, and temporal shifts in patient-physician relationships over the time frames examined could also impact the result differences (2012 vs 2012-2018 in our data).

### Implications

Our findings have important implications for health care researchers, professionals, and policy makers. First, the empirical evidence of web-based reviews is less favorable toward female physicians, after accounting or controlling for several clinical aspects (including specialty and Medicare patient risk), and temporal effects should inform health care professionals and policy makers that patients’ opinions are consistently more favorable toward male physicians than toward female physicians. This cannot be overlooked even though the magnitude of the effect of gender on web-based reviews is not sizable.

### Policy and Design Suggestions

Gender bias in reviews has been reported across multiple domains, including academia. Murray et al [66] found that male faculty tended to receive higher ratings for overall teaching quality than female faculty, and Turrentine et al [67] and Rojek et al [68] found implicit bias in the narrative evaluations, with a bias toward men receiving more superlative praise. Studies have shown that measures can be taken to help reduce gender bias in reviews. Peterson et al [69] found that simply informing students of potential gender biases can have significant effects on the evaluation of female instructors, and Rivera and Tilcsik [70] found that by changing the rating scale from a 10-point to a 6-point rating system, gender bias can be reduced.

Large societal-level aspects may also be in effect; however, that would seemingly be very hard to account for within a single portal. Sprague and Massoni [71] found that male teachers are more likely to be held to an entertainer standard, whereas female teachers are held to a nurturer standard. These biases are formed throughout an individual’s life, and therefore, are harder to adjust for, even when directly informing users of the potential for bias. By leveraging the lessons learned from gender bias studies, web-based physician review sites could help to mitigate, but not eliminate, gender bias within their systems.

Concentrated efforts to educate and inform patients about female physicians’ competence are needed. This can help to reduce implicit bias among patients toward the competence of female physicians compared with their male counterparts. These websites serve as an important resource for both reviewers and readers of the reviews, and the information needs to flow well. At the same time, readers of the reviews may be served better if the reviewers are asked to provide opinions about physicians of different genders before they provide a review for a physician. To solicit reviewers’ predisposed opinions about physicians of different genders, the questions can be framed in a manner that does not make the reviewers feel that they are being investigated for their opinions. After collecting their opinions on this issue, the websites may consider filtering the reviews provided by reviewers with an overt bias against physicians of one gender. The question of how to design the website to reduce the possible gender bias is complex and requires serious thought and consideration from both researchers and website designers. By leveraging previous research efforts targeted at informing users of bias potential, review portals can better collect and present information about physicians.

### Limitations

Our study has a few limitations. First, we constructed our patient risk scores using the HCC risk score from Medicare data. Although Medicare is among the largest health care payers or insurers in the United States, further studies can attempt to validate the findings of our study using clinical data from other insurers. For instance, a significant proportion of the patient population in the United States has insurance from private insurers. Future studies can attempt to validate our findings by constructing clinical variables, such as risk scores, using clinical data from one or more private insurers. Second, we focused on the physician data from Alabama. Although it is 1 state, it provides a good mix of rural and urban counties. Future studies could extend this work to other states and compare the findings across a broader set of patients and health care providers.

### Future Studies

The findings of this study suggest that gender bias in web-based reviews needs to be examined more closely. Additional studies that identify factors impacting this gender bias could help us develop strategies to mitigate gender bias in web-based reviews. Given the shortage of health care providers and the need for a robust and diverse health care workforce, such studies can help not only the service providers but also policy makers, educators, and administrators. If the administrators of hospitals and clinics are made aware of this bias and acknowledge it accordingly, institutional changes can be implemented to support and empower women to take up more leadership roles in clinical settings. As Sandberg [72] points out in her New York Times best seller, as fewer women are in leadership roles than men, it can be challenging for junior women to have mentorship opportunities. A possible solution to this problem could be the performance evaluations of male leadership personnel to include the number of women mentored and focused initiatives and incentive opportunities for women to take on pathways to leadership roles.

These focused efforts can provide a strong signal to patients about the competence of female physicians and, in turn, increase their confidence in the care provided by female physicians. This can further help to improve the overall care delivered to patients, as the increase in patients’ confidence can improve their communication with physicians, irrespective of the physicians’ gender. However, an open research question is whether the bias observed in web-based physician reviews is also observable in offline physician surveys. To examine this question, studies that compare reviews of male and female physicians in web-based and offline media need to be conducted.

## Appendix

Multimedia Appendix 1 Tables depicting the results of additional analysis including robustness checks.

## Abbreviations

CMS: Center for Medicare and Medicaid Services
HCC: hierarchical condition category
LDA: Latent Dirichlet Allocation
