# Supplementary material for: Physician Gender, Patient Risk, and Web-Based Reviews: Longitudinal Study of the Relationship Between Physicians’ Gender and Their Web-Based Reviews
Source: J Med Internet Res. 2022 Apr 8;24(4):e31659. doi: 10.2196/31659 (PMC9034420; doi:10.2196/31659)
Supplement: Multimedia Appendix 1 [file jmir_v24i4e31659_app1.docx]

## Multimedia Appendix 1

| **Table S1.** Count of Physicians by Gender across Specialties | | | | | | |
| --- | --- | --- | --- | --- | --- | --- |
| **Specialty** | | **Male Physician Count** | | **Female Physician Count** | | |
| allergy | | 3 | | 4 | | |
| anesthesiology | | 5 | | 0 | | |
| cardio thoracic surgery | | 1 | | 0 | | |
| cardiology | | 36 | | 6 | | |
| chiropractic | | 17 | | 1 | | |
| dentistry | | 1 | | 0 | | |
| dermatology | | 19 | | 18 | | |
| emergency medicine | | 8 | | 0 | | |
| endocrinology | | 17 | | 7 | | |
| gastroenterology | | 18 | | 2 | | |
| general (family) practice | | 135 | | 76 | | |
| general surgery | | 28 | | 4 | | |
| hematology and oncology | | 10 | | 4 | | |
| infectious disease | | 1 | | 0 | | |
| internal medicine | | 80 | | 27 | | |
| nephrology | | 5 | | 0 | | |
| neuro surgery | | 13 | | 0 | | |
| neurology | | 39 | | 8 | | |
| nursing | | 1 | | 6 | | |
| obstetrics and gynecology | | 111 | | 71 | | |
| ophthalmology and optometry | | 32 | | 9 | | |
| orthopedic surgery | | 48 | | 4 | | |
| otolaryngology | | 42 | | 3 | | |
| pain management | | 8 | | 1 | | |
| plastic surgery | | 17 | | 0 | | |
| podiatry | | 24 | | 5 | | |
| proctology | | 1 | | 0 | | |
| psychiatry | | 32 | | 7 | | |
| psychology | | 4 | | 4 | | |
| pulmonology | | 17 | | 1 | | |
| radiology | | 1 | | 0 | | |
| rheumatology | | 18 | | 5 | | |
| urology | | 22 | | 4 | | |
| vascular surgery | | 2 | | 0 | | |
| Total | | 816 | | 277 | | |
| **Table S2.** Estimation for *OverallRating* with Additional Controls (N=1093)^a^ | | | | | |  |
| Variable | Coefficient | | SE | | *p* value |  |
| *GenderFemale* | -0.194 | | 0.060 | | .001 |  |
| *RiskScore* | -0.069 | | 0.087 | | .43 |  |
| *TopicCareYear* | 1.430 | | 0.061 | | <.001 |  |
| *TopicSurgeryYear* | 0.733 | | 0.071 | | <.001 |  |
| *BeneficiaryCount* | -0.000 | | 0.000 | | .004 |  |
| *ServicesCount* | 0.000 | | 0.000 | | .91 |  |
| *WordCount* | -0.003 | | 0.000 | | <.001 |  |
| ^a^Specialty controls=yes; year fixed effects=yes; robust SE=yes; overall R-squared=0.288; within R-squared=0.183; between R-squared=0.364. | | | | | |  |

| **Table S3.** Estimation for *HelpfulnessRating* and *KnowledgeRating* with Additional Controls (N=1093) | | | | | | |
| --- | --- | --- | --- | --- | --- | --- |
| Variable | *HelpfulnessRating*^a^ | | | *KnowledgeRating*^b^ | | |
|  | Coefficient | SE | *p* value | Coefficient | SE | *p* value |
| *GenderFemale* | -0.221 | 0.069 | .001 | -0.230 | 0.065 | <.001 |
| *RiskScore* | -0.016 | 0.099 | .87 | -0.072 | 0.094 | .45 |
| *TopicCare* | 1.539 | 0.072 | <.001 | 1.352 | 0.066 | <.001 |
| *TopicSurgery* | 0.680 | 0.083 | <.001 | 0.506 | 0.079 | <.001 |
| *BeneficiaryCount* | -0.000 | 0.000 | .003 | -0.000 | 0.000 | .004 |
| *ServicesCount* | 0.000 | 0.000 | .96 | 0.000 | 0.000 | .72 |
| *WordCount* | -0.004 | 0.000 | <.001 | -0.004 | 0.000 | <.001 |
| ^a^Specialty controls=yes; year fixed effects=yes; robust SE=yes; overall R-squared=0.263; within R-squared=0.172; between R-squared=0.337. | | | | | | |
| ^b^Specialty controls=yes; year fixed effects=yes; robust SE=yes; overall R-squared=0.241; within R-squared=0.152; between R-squared=0.307. | | | | | | |

| **Table S4.** Estimation for *StaffRating* and *PunctualityRating* with Additional Controls (N=1093) | | | | | | |
| --- | --- | --- | --- | --- | --- | --- |
| Variable | *StaffRating*^a^ | | | *PunctualityRating*^b^ | | |
|  | Coefficient | SE | *p* value | Coefficient | SE | *p* value |
| *GenderFemale* | -0.123 | 0.062 | .049 | -0.200 | 0.067 | .003 |
| *RiskScore* | -0.051 | 0.088 | 0.56 | -0.139 | 0.106 | .19 |
| *TopicCare* | 1.447 | 0.066 | <.001 | 1.380 | 0.066 | <.001 |
| *TopicSurgery* | 0.918 | 0.076 | <.001 | 0.827 | 0.074 | <.001 |
| *BeneficiaryCount* | -0.000 | 0.000 | .01 | -0.000 | 0.000 | .02 |
| *ServicesCount* | 0.000 | 0.000 | .89 | 0.000 | 0.000 | .81 |
| *WordCount* | -0.003 | 0.000 | <.001 | -0.003 | 0.000 | <.001 |
| ^a^Specialty controls=yes; year fixed effects=yes; robust SE=yes; overall R-squared=0.259; within R-squared=0.163; between R-squared=0.331. | | | | | | |
| ^b^Specialty controls=yes; year fixed effects=yes; robust SE=yes; overall R-squared=0.249; within R-squared=0.139; between R-squared=0.337. | | | | | | |

| **Table S5.** Estimation for *OverallRating* with Both Genders in Specialties (N=1038)^a^ | | | |
| --- | --- | --- | --- |
| Variable | Coefficient | SE | *p* value |
| *GenderFemale* | -0.198 | 0.060 | .001 |
| *RiskScore* | -0.112 | 0.088 | .20 |
| *TopicCareYear* | 1.427 | 0.062 | <.001 |
| *TopicSurgeryYear* | 0.714 | 0.073 | <.001 |
| *BeneficiaryCount* | -0.000 | 0.000 | .003 |
| *Number of Services* | 0.000 | 0.000 | .90 |
| *WordCountYear* | -0.003 | 0.000 | <.001 |
| ^a^Specialty controls=yes; year fixed effects=yes; robust SE=yes; overall R-squared=0.288; within R-squared=0.184; between R-squared=0.362. | | | |

| **Table S6.** Estimation for *HelpfulnessRating* and *KnowledgeRating* with Both Genders in Specialties (N=1038) | | | | | | |
| --- | --- | --- | --- | --- | --- | --- |
| Variable | *HelpfulnessRating*^a^ | | | *KnowledgeRating*^b^ | | |
|  | Coefficient | SE | *p* value | Coefficient | SE | *p* value |
| *GenderFemale* | -0.226 | 0.069 | .001 | -0.233 | 0.065 | <.001 |
| *RiskScore* | -0.066 | 0.103 | .52 | -0.102 | 0.096 | .29 |
| *TopicCareYear* | 1.544 | 0.073 | <.001 | 1.352 | 0.067 | <.001 |
| *TopicSurgeryYear* | 0.666 | 0.085 | <.001 | 0.494 | 0.081 | <.001 |
| *BeneficiaryCount* | -0.000 | 0.000 | .003 | -0.000 | 0.000 | .003 |
| *Number of Services* | 0.000 | 0.000 | .95 | 0.000 | 0.000 | .69 |
| *WordCountYear* | -0.004 | 0.000 | <.001 | -0.004 | 0.000 | <.001 |
| ^a^Specialty controls=yes; year fixed effects=yes; robust SE=yes; overall R-squared=0.263; within R-squared=0.172; between R-squared=0.335. | | | | | | |
| ^b^Specialty controls=yes; year fixed effects=yes; robust SE=yes; overall R-squared=0.241; within R-squared=0.154; between R-squared=0.306. | | | | | | |

| **Table S7.** Estimation for *StaffRating* and *PunctualityRating* with Both Genders in Specialties (N=1038) | | | | | | |
| --- | --- | --- | --- | --- | --- | --- |
| Variable | *StaffRating*^a^ | | | *PunctualityRating*^b^ | | |
|  | Coefficient | SE | *p* value | Coefficient | SE | *p* value |
| *GenderFemale* | -0.128 | 0.062 | .04 | -0.205 | 0.067 | .002 |
| *RiskScore* | -0.088 | 0.091 | .33 | -0.198 | 0.108 | .07 |
| *TopicCareYear* | 1.434 | 0.067 | <.001 | 1.375 | 0.067 | <.001 |
| *TopicSurgeryYear* | 0.884 | 0.079 | <.001 | 0.809 | 0.076 | <.001 |
| *BeneficiaryCount* | -0.000 | 0.000 | .008 | -0.000 | 0.000 | .02 |
| *Number of Services* | 0.000 | 0.000 | .85 | -0.000 | 0.000 | .76 |
| *WordCountYear* | -0.003 | 0.000 | <.001 | -0.003 | 0.000 | <.001 |
| ^a^Specialty controls=yes; year fixed effects=yes; robust SE=yes; overall R-squared=0.257; within R-squared=0.162; between R-squared=0.326. | | | | | | |
| ^b^Specialty controls=yes; year fixed effects=yes; robust SE=yes; overall R-squared=0.245; within R-squared=0.137; between R-squared=0.333. | | | | | | |

| **Table S8.** Estimation for *OverallRating* without Topic Controls (N=1093)^a^ | | | |
| --- | --- | --- | --- |
| Variable | Coefficient | SE | *p* value |
| *GenderFemale* | -0.180 | 0.070 | 0.01 |
| *RiskScore* | -0.057 | 0.097 | 0.556 |
| ^a^Specialty controls=yes; year fixed effects=yes; robust SE=yes; overall R-squared=0.080; within R-squared=0.005; between R-squared=0.132. | | | |

**Table S9.** Estimation for *HelpfulnessRating* and *KnowledgeRating* without Topic Controls (N=1093)

| Variable | *HelpfulnessRating*^a^ | | | *KnowledgeRating*^b^ | | |
| --- | --- | --- | --- | --- | --- | --- |
|  | Coefficient | SE | *p* value | Coefficient | SE | *p* value |
| *GenderFemale* | -0.202 | 0.079 | 0.01 | -0.211 | 0.074 | 0.004 |
| *RiskScore* | 0.001 | 0.110 | 0.993 | -0.062 | 0.104 | 0.553 |
| ^a^Specialty controls=yes; year fixed effects=yes; robust SE=yes; overall R-squared=0.072; within R-squared=0.005; between R-squared=0.127. | | | | | | |
| ^b^Specialty controls=yes; year fixed effects=yes; robust SE=yes; overall R-squared=0.069; within R-squared=0.003; between R-squared=0.118. | | | | | | |

| **Table S10.** Estimation for *StaffRating* and *PunctualityRating* without Topic Controls (N=1093) | | | | | | | |
| --- | --- | --- | --- | --- | --- | --- | --- |
| Variable | *StaffRating*^a^ | | | *PunctualityRating*^b^ | | |  |
|  | Coefficient | SE | *p* value | Coefficient | SE | *p* value |  |
| *GenderFemale* | -0.118 | 0.071 | 0.093 | -0.190 | 0.077 | 0.013 |  |
| *RiskScore* | -0.048 | 0.098 | 0.627 | -0.126 | 0.112 | 0.262 |  |
| ^a^Specialty controls=yes; year fixed effects=yes; robust SE=yes; overall R-squared=0.081; within R-squared=0.004; between R-squared=0.135. | | | | | | |  |
| ^b^Specialty controls=yes; year fixed effects=yes; robust SE=yes; overall R-squared=0.068; within R-squared=0.005; between R-squared=0.112. | | | | | | |  |

| **Table S11.** Descriptive Statistics for Original RateMDs and Medicare Data | | | | |
| --- | --- | --- | --- | --- |
| Variable | Values, mean (SD) | Values, median | Values, minimum | Values, maximum |
| *OverallRating* | 3.82 (1.30) | 4.38 | 1 | 5 |
| *HelpRating* | 3.75 (1.50) | 4.5 | 1 | 5 |
| *KnowledgeRating* | 3.91 (1.40) | 4.86 | 1 | 5 |
| *StaffRating* | 3.86 (1.33) | 4.33 | 1 | 5 |
| *PunctualityRating* | 3.75 (1.35) | 4 | 1 | 5 |
| *TopicCare* | 0.43 (0.41) | 0.4 | 0 | 1 |
| *TopicSurgery* | 0.27 (0.37) | 0 | 0 | 1 |
| *TopicStaff* | 0.30 (0.38) | 0 | 0 | 1 |
| *RiskScore* | 1.49 (0.72) | 1.3 | 0.44 | 10.66 |
